# Transcriptome Profiling of *Euproctis pseudoconspersa* Reveals Candidate Olfactory Genes for Type III Sex Pheromone Detection

**DOI:** 10.3390/ijms26041405

**Published:** 2025-02-07

**Authors:** Xiangzhi Zhang, Shunsi Li, Zongxiu Luo, Xiaoming Cai, Lei Bian, Chunli Xiu, Nanxia Fu, Naiyong Liu, Zhengqun Zhang, Zhaoqun Li

**Affiliations:** 1 National Key Laboratory for Tea Plant Germplasm Innovation and Resource Utilization, Tea Research Institute, Chinese Academy of Agricultural Science, Hangzhou 310008, China; zxz19991221@163.com (X.Z.);; 2 College of Horticulture Science and Engineering, Shandong Agricultural University, Taian 271000, China; 3 Key Laboratory of Forest Disaster Warning and Control of Yunnan Province, Southwest Forestry University, Kunming 650224, China

**Keywords:** transcriptomic profiling, olfactory gene, *Euproctis pseudoconspersa*, type III sex pheromone

## Abstract

The tea tussock moth (*Euproctis pseudoconspersa*) is a common tea plantation pest with Type III sex pheromone components (SPCs). However, the olfactory genes involved in the perception of Type III SPCs remain unknown. To identify the olfactory genes involved in *E*. *pseudoconspersa* olfactory perception, we sequenced the transcriptomes of different tissues from male and female moths. We identified 27 chemosensory proteins, 39 odorant-binding proteins (OBPs), 28 ionotropic receptors (IRs), and 67 odorant receptors (ORs). Phylogenetic and antennal abundance analyses showed that EpseOR12, EpseOR13, EpseOR15, EpseOR16, and EpseOR18 belonged to the pheromone receptor clades of Type II moths, with predominant expression in male antennae. Besides these EpseORs, EpseOR14 and EpseOR32 were two of the most abundant EpseORs in male antennae, where they were predominantly expressed. Four pheromone-binding proteins (PBPs) were identified, with higher expression in male antennae. EpseORs and EpsePBPs may be involved in Type III SPC detection. Additionally, a few EpseOBPs, EpseIRs, and EpseORs were predominantly expressed in either male or female antennae. These genes may play important roles in olfaction and may be involved in detecting host plant volatiles and pheromones. These results provide a foundation for further exploration of the molecular mechanisms of *E*. *pseudoconspersa* olfaction.

## 1. Introduction

Insects rely on sophisticated olfactory systems to complete important physiological behaviors, such as locating food, mating partners, and oviposition sites [1,2]. To adapt to diverse environments and ecological niches, insects have evolved unique and efficient olfactory systems to perceive crucial chemical signals, such as sex pheromone components (SPCs) and plant volatiles [3,4]. Therefore, understanding the olfactory molecular mechanisms of insects may provide new insights for devising pest control strategies. The olfactory process in insects involves various olfactory proteins, such as chemosensory proteins (CSPs), odorant-binding proteins (OBPs), odorant receptors (ORs), and ionotropic receptors (IRs) [2]. Among these, OBPs and CSPs bind and transport odor molecules through the sensillar lymph to olfactory receptors [5,6,7], whereas ORs and IRs mediate the transduction of odor molecules from chemical information into electric signals [8].

In lepidopterans, SPCs are secreted by female moths for interspecies communication [3,9]. Since the initial identification of SPCs in silkworms by Butenandt et al. in 1959, synthetic SPCs have been used extensively in pest management [10]. Moth SPCs can be classified into four types according to their chemical structure: Type I, II, III, and 0 [3,11]. Type I SPCs are unsaturated compounds with straight hydrocarbon chains and oxygenated functional groups, such as ester linkages, alcohols, and aldehydes. Type II pheromones comprise straight hydrocarbons with multiple double bonds and epoxide derivatives. Type III SPCs typically contain monomethyl- or dimethyl-branched long-chain hydrocarbons, including a chiral center. Finally, Type 0 SPCs are proposed moieties containing short-chain methylcarbinols and methylketones.

As SPCs are special chemical signals, OBPs and ORs that detect SPCs are named pheromone-binding proteins (PBPs) and pheromone receptors (PRs), respectively [12,13,14,15]. Lepidopteran PBPs and PRs constitute a subfamily of insect OBPs and ORs, respectively. All SPC detection mechanisms in lepidopteran insects, such as *Bombyx mori* and *Helicoverpa armigera*, have focused primarily on Type I and II pheromones [16]. Because of the rarity of Type III pheromone lepidopteran insects, no PBPs or PRs responsive to Type III pheromones have been found [3]. Therefore, whether moths evolve different types of PBPs and PRs to match the different types of pheromones or whether the existing receptors are adapted to detect different types of pheromones remains unknown.

The tea tussock moth, *Euproctis pseudoconspersa,* is a destructive chewing pest found in tea plantations in China, Japan, and Korea [17]. The larvae damage the leaves and result in heavy losses in the quantity and quality of tea produced. Furthermore, venomous spicules on the back are hazardous to the human skin, causing severe allergic reactions and skin inflammation. The SPCs of *E. pseudoconspersa* have been identified as 10,14-dimethyl-pentadecyl isobutyrate (10Me14Me-15:iBu) and 14-methylpentadecyl isobutyrate (14Me-15:iBu), which are typical Type III SPCs [17,18]. Our previous studies showed that 10Me14Me-15:iBu is a chiral branched methyl compound, with the (*R*)-enantiomer exhibiting a significantly stronger electroantennogram response and field attractiveness than the (S)-enantiomer [19]. However, no information on olfactory genes is available for *E. pseudoconspersa*. Obtaining sequence data is important in the study of olfactory perception mechanisms, including pheromone detection. Therefore, the current study conducted tissue transcriptome profiling of *E. pseudoconspersa* and analyzed phylogeny, tissue abundance, and tissue expression patterns to identify olfactory-related genes.

## 2. Results

### 2.1. Transcriptome Overview and Identification of EpseCSPs, EpseOBPs, EpseIRs, and EpseORs

Tissue transcriptome profiles of *E. pseudoconspersa* were sequenced using the Illumina platform. Overall, 21 samples were sequenced, and approximately 1.22 billion clean reads (183.38 Gb data) were obtained. Transcriptomic data were available from the National Center for Biotechnology Information (NCBI) Sequence Read Archive database (http://trace.ncbi.nlm.nih.gov/Traces/sra/; accession numbers: SRR31971029-SRR31971049, accessed on 13 January 2025.). After filtering and assembling, 295,509 unigenes were obtained, with an N_50_ length of 2002 nt (Table S1). For annotation, the unigenes were searched against the Nr, Nt, Pfam, KOG/COG, Swiss-prot, KEGG, and GO databases using BLASTX(v2.2.28+) with a cut-off *e*-value of 10^−5^. In total, 169,459 (57.34%) unigenes were annotated using at least one database. Of these, 106,311 (35.97%) genes were successfully annotated in the Nr database (Table S2). Sequence annotation and analysis revealed 27 EpseCSPs, 39 EpseOBPs, 28 EpseIRs, and 67 EpseORs (Table S3). Of these, 25 EpseCSPs, 35 EpseOBPs, 15 EpseIRs, and 56 EpseORs contained full-length open reading frames (ORFs).

### 2.2. Phylogenetic Analysis of E. pseudoconspersa OBPs, ORs, and IRs

**Figure 1:**
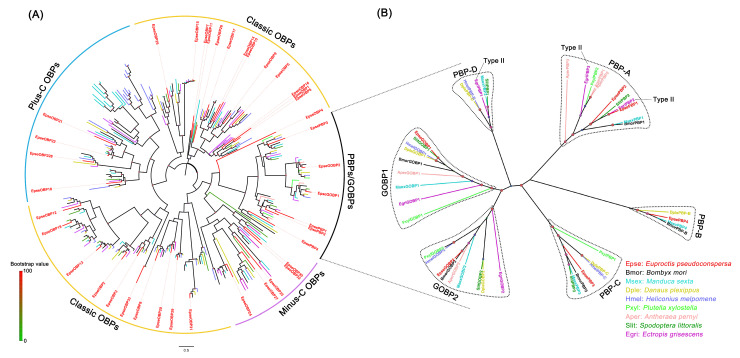
Phylogenetic tree of EpseOBPs with other lepidopteran insects: (**A**) phylogenetic analysis of all EpseOBPs with other lepidopteran insects; and (**B**) phylogenetic tree of EpsePBPs and EpseGOBPs with PBPs and GOBPs of other lepidopteran insects. The color of dots on the branches shows the bootstrap values. Type II, PBPs for Type II SPCs. The phylogenetic tree was constructed in PhyML 3.1 using the maximum-likelihood method.

To further investigate the potential function of *E. pseudoconspersa* OBP, OR, and IR genes, phylogenetic analyses were performed using the amino acid sequences of *E. pseudoconspersa* and other lepidopteran insects, including Type I and II pheromone species. EpseOBPs were scattered across different subfamilies, such as classic, minus-C, and plus-C OBPs (Figure 1). Two EpseGOBPs and four EpsePBPs were well clustered in the PBP/GOBP subgroup. EpseGOBP1 and EpseGOBP2 were distributed within the GOBP clade. EpsePBPs were distributed in the PBP clade. EpsePBP1 and EpsePBP2 belonged to the PBP-A group, EpsePBP4 belonged to the PBP-B group, and EpsePBP3 belonged to the PBP-C group.

**Figure 2:**
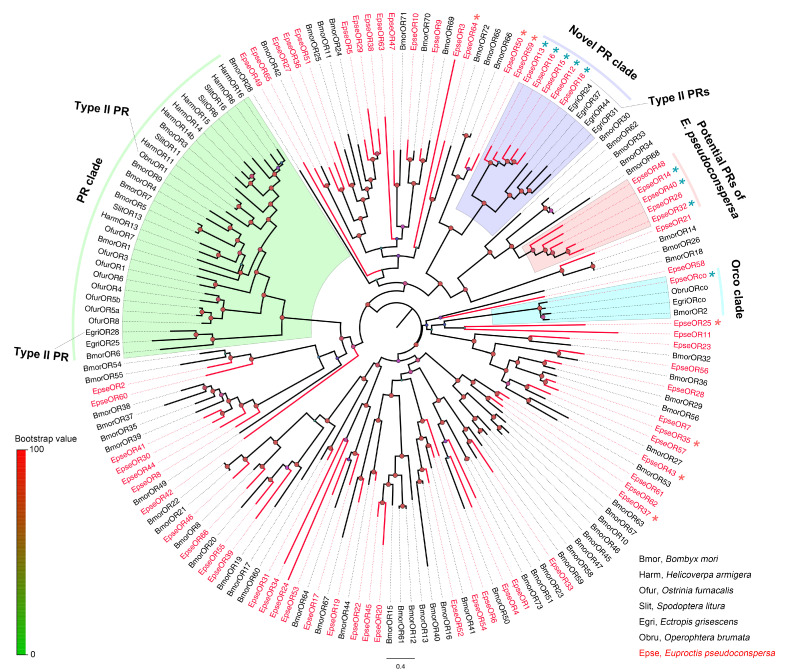
Phylogenetic tree of EpseORs with other lepidopteran insects. The phylogenetic tree was constructed in PhyML 3.1 using the maximum-likelihood method. The color of dots on the branches shows the bootstrap values. The group in blue was classified as “Orco”. The group in green was classified as the “PR clade”. The group in purple was classified as the “Novel PR clade”. The group in red was classified as “putative PRs of *E. peseudoconspersa*”. Blue *, *EpseORs* were significantly more abundant in male than female antennae. Red *, *EpseORs* were significantly more abundant in female than male antennae. *p*-value < 0.05.

**Figure 3:**
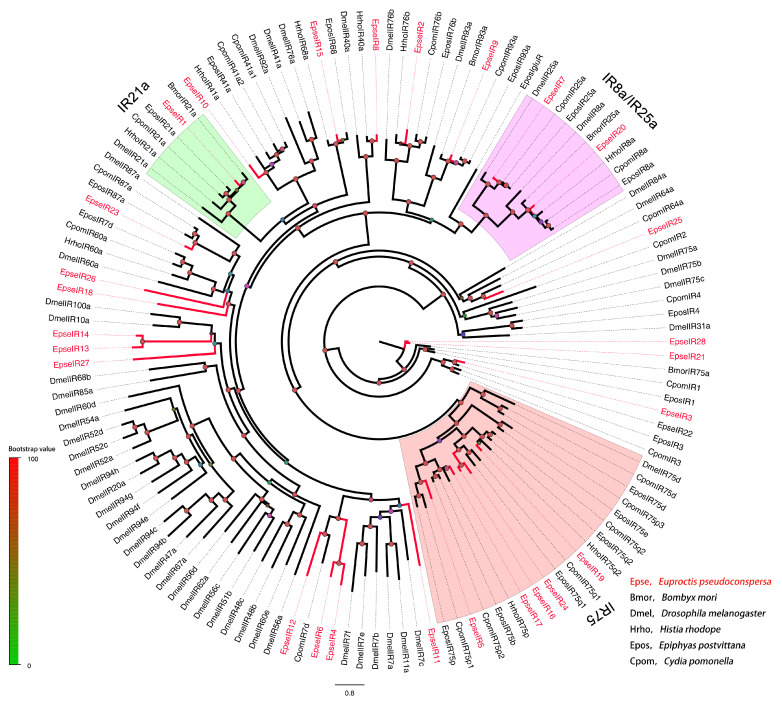
Phylogenetic tree of EpseIRs with other lepidopteran insects. The phylogenetic tree was constructed in PhyML 3.1 using the maximum-likelihood method. The color of dots on the branches shows the bootstrap values. The group in green was classified as “IR21a”. The group in pink was classified as “IR8a/IR25A”. The group in red was classified as “IR75”.

In the OR phylogenetic tree, EpseORco was clustered well within the OR co-receptors (ORcos) of Type I and II pheromone insects (Figure 2). No EpseORs clustered in the PR clade, which consisted of Type I and II PRs. Five EpseORs (EpseOR12, EpseOR13, EpseOR15, EpseOR16, and EpseOR18) were distributed in a novel PR clade with the PRs for Type II SPC detection. These EpseORs might have been PRs of *E. pseudoconspersa*. However, five EpseORs—EpseOR14, EpseOR26, EpseOR32, EpseOR40, and EpseOR48—formed a separate subgroup without orthologs in other lepidopteran insects. The IR phylogenetic tree showed that EpseIR20 was distributed in the IR8a/IR25a clade (Figure 3). EpseIR1 and EpseIR8 clustered in the IR21a and IR40a groups, respectively. Ten EpseIRs, including EpseIR3, EpseIR5, EpseIR16, EpseIR17, EpseIR19, EpseIR21, EpseIR22, EpseIR24, EpseIR25, and EpseIR28, were grouped into the IR79 subfamily.

### 2.3. Abundance of Olfactory Receptor Genes

**Figure 4:**
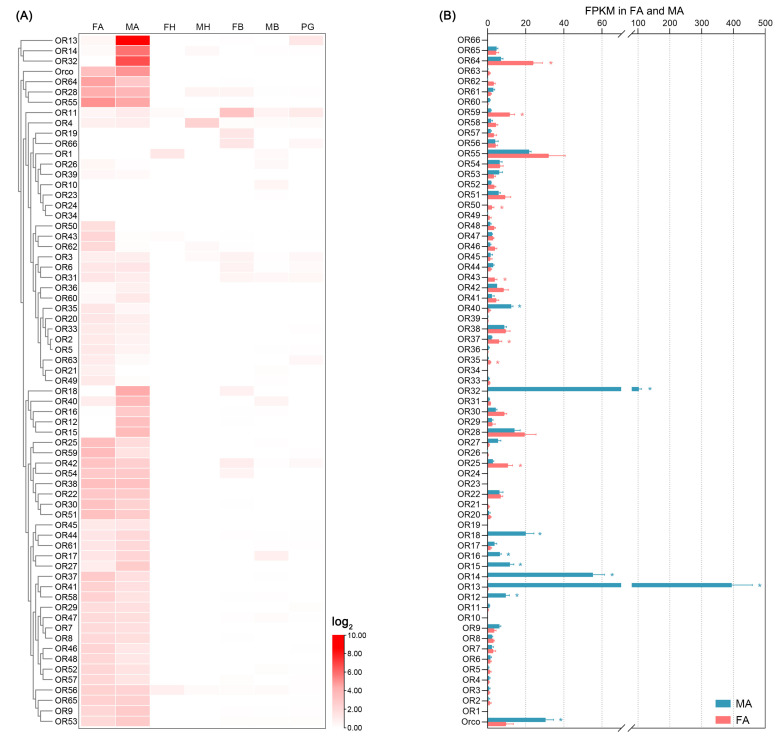
Abundance of *EpseOBPs* in transcriptome profiling of male and female *Euproctis pseudoconspersa*: (**A**) abundance of *EpseOBPs* in the antennae, heads, bodies, and pheromone glands of male and female *E. pseudoconspersa*; and (**B**) FKPM values of *EpseOBPs* in *E. pseudoconspersa* antennae. FA, female antenna; MA, male antenna; FH, female head without antennae; MH, male head without antennae; FB, female body without antennae, head, and pheromone glands; MB, male body without antennae and head; and PG, pheromone gland. Blue *, *EpseOBPs* were significantly more abundant in male than females antennae. Red *, *EpseOBPs* were significantly more abundant in female than male antennae. *p*-value < 0.05.

**Figure 5:**
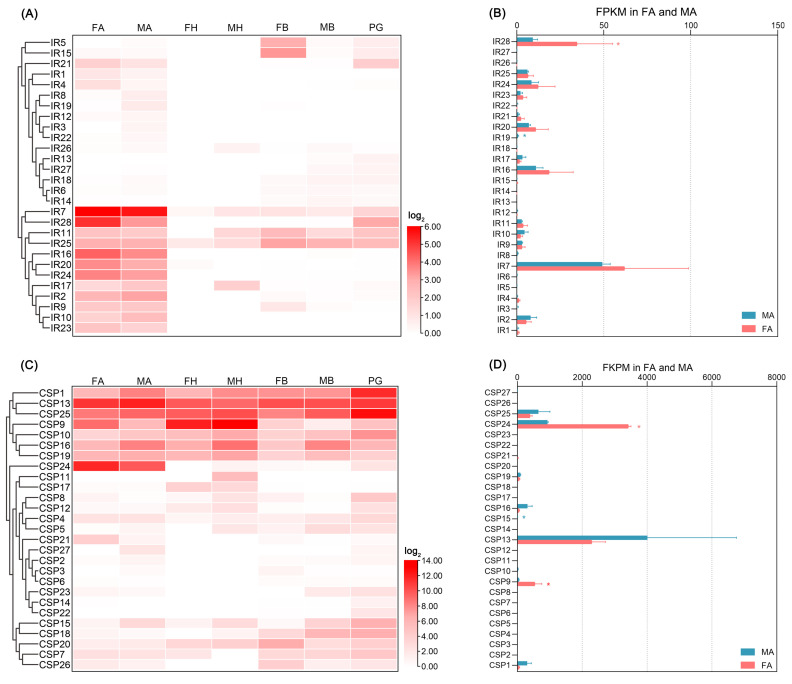
Abundance of *EpseCSPs* and *EpseIRs* in transcriptome profiling of male and female *Euproctis pseudoconspersa*: (**A**) abundance of *EpseCSPs* in the antennae, heads, bodies, and pheromone glands of male and female *E. pseudoconspersa*; (**B**) FKPM values of *EpseCSPs* in *E. pseudoconspersa* antennae; (**C**) abundance of *EpseIRs* in the antennae, heads, bodies, and pheromone glands of male and female *E. pseudoconspersa*; and (**D**) FKPM values of *EpseIRs* in *E. pseudoconspersa* antennae. FA, female antenna; MA, male antenna; FH, female head without antennae; MH, male head without antennae; FB, female body without antennae, head, and pheromone glands; MB, male body without antennae and head; and PG, pheromone gland. Blue *, genes were significantly more abundant in male than female antennae. Red *, genes were significantly more abundant in female than male antennae. *p*-value < 0.05.

To investigate the expression profiles of *E. pseudoconspersa* olfactory genes, abundance analyses of these genes were constructed by evaluating their fragments per kilobase per million mapped reads (FPKM) values. Most *EpseORs* and *EpseIRs* were uniquely or predominantly expressed in the antennae (Figure 4A and Figure 5A). Although several *EpseORs* had high antenna mRNA abundance with FPKMs > 100, the FPKM of most *EpseORs* and all *EpseIRs* was lower than 20 (Table S4). Of the 67 *EpseORs*, *EpseORco*, *EpseOR12*, *EpseOR13*, *EpseOR14*, *EpseOR15*, *EpseOR16*, *EpseOR18*, *EpseOR32*, and *EpseOR40* were significantly more highly expressed in the antennae of males than females (Figure 4B). Of these, *EpseOR14*, *EpseOR32*, and *EpseOR13* were the three most abundant EpseORs in male antennae, with FPKM values of 394.8, 102.8, and 50.2, respectively. Therefore, these three EpseORs may have functioned as PRs. Seven *EpseORs* (*EpseOR25*, *EpseOR35*, *EpseOR37*, *EpseOR46*, *EpseOR50*, *EpseOR59*, and *EpseOR64*) were more strongly expressed in female antennae than in male antennae. *EpseIR7* and *EpseIR28* were the two most abundant *EpseIRs* in antennae (Figure 5B). *EpseIR28* had significantly higher antenna mRNA abundance in females than in males. The FPKM value of *EpseIR19* was significantly higher in males than in females.

### 2.4. Abundance of EpseOBP and EpseCSP Genes

**Figure 6:**
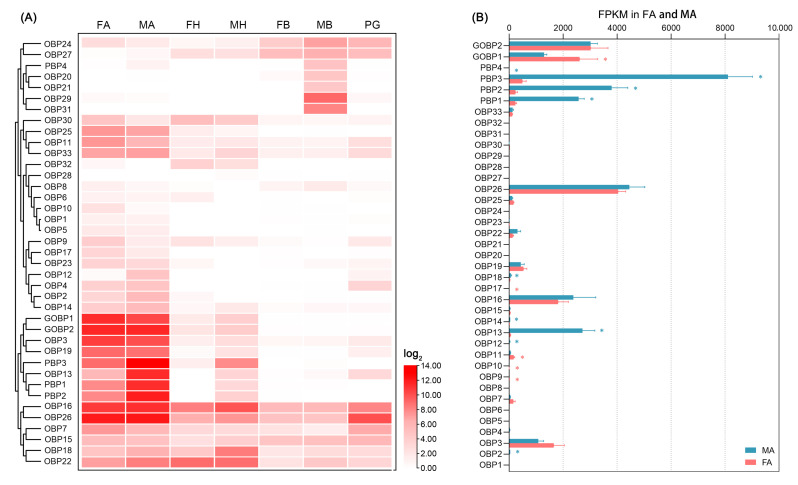
Abundance of *EpseORs* in transcriptome profiling of male and female *Euproctis pseudoconspersa*: (**A**) abundance of *EpseORs* in the antennae, heads, bodies, and pheromone glands of male and female *E. pseudoconspersa*; and (**B**) FKPM values of *EpseORs* in *E. pseudoconspersa* antennae. FA, female antenna; MA, male antenna; FH, female head without antennae; MH, male head without antennae; FB, female body without antennae, head, and pheromone glands; MB, male body without antennae and head; and PG, pheromone gland. Blue *, *EpseORs* were significantly more abundant in male than female antennae. Red *, *EpseORs* were significantly more abundant in female than male antennae. *p*-value < 0.05.

*EpseOBPs* and *EpseCSPs* had higher FPKM values than *EpseORs* and *EpseIRs* (Table S4). Most *EpseOBPs* exhibited antenna-biased expression (Figure 6A). Of these, *EpseOBP2*, *EpseOBP12*, *EpseOBP13*, *EpsePBP1*, *EpsePBP2*, and *EpsePBP3* showed significantly higher transcript levels in male than female antennae (Figure 6B). Of these male antenna-biased *EpseOBPs*, *EpsePBP1*, *EpsePBP2*, and *EpsePBP3* were much more abundant in male antennae, with FPKM values of 2577.0, 3799.5, and 8110.3, respectively. The FPKM value of *EpseOBP13* in male antennae was 2721.2, much higher than that of other male antenna-biased *EpseOBPs* (FPKM < 50). Five *EpseOBPs*—*EpseOBP9*, *EpseOBP10*, *EpseOBP11*, *EpseOBP17*, and *EpseGOBP1*—were significantly more abundant in female antennae than in male antennae. Of these, *EpseGOBP1* (FPKM: 2610.3) was the most abundant *EpseOBP* in female antennae. *EpseGOBP2* showed higher FPKM values in both female and male antennae. *EpseCSPs* were ubiquitous in most tissues at relatively high levels (Figure 5C). Among the antenna-biased *EpseCSPs*, *EpseCSP24* was significantly highly expressed in female antennae, with a higher FPKM than the rest, at 3422.0 (Figure 5D).

### 2.5. qPCR Validation

**Figure 7:**
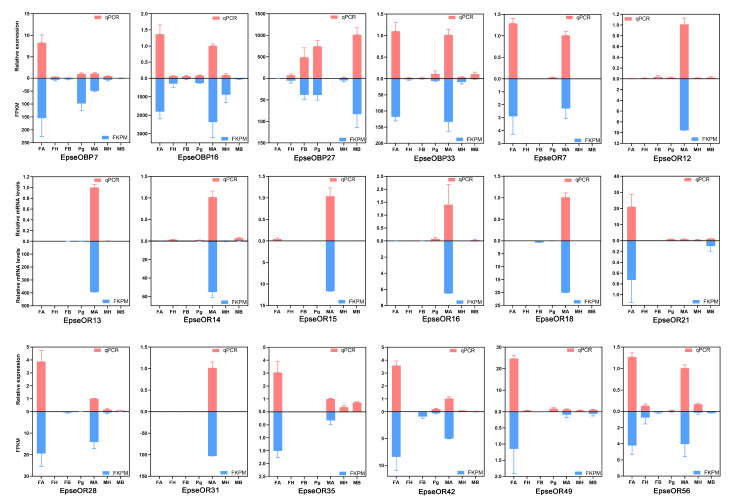
Verification of 18 selected olfactory genes in the antennae, heads, bodies, and pheromone glands of male and female *E. pseudoconspersa*. Red, qPCR results; and blue, FKPM data. Error bars represent the standard error.

According to the tissue transcriptome profile data, most *EpseOBPs* and *EpseORs* were abundant in the antennae. To verify the abundance of the tissue transcriptome profiles, 8 genes, including four *EpseOBPs* and 14 *EpseORs*, were selected to examine the relative expression levels. The qPCR results for these genes were consistent with those of tissue transcriptome profiles (Figure 7). Of these EpseORs, EpseOR12, EpseOR13, EpseOR14, EpseOR15, EpseOR16, EpseOR18, and EpseOR32 were exclusively or preferentially expressed in antennae, with male-biased expression.

## 3. Discussion

SPCs play key roles in insect reproductive isolation. Each lepidopteran species produces specific SPCs with different chemical structures or a diverse blend of components. The highly varied lepidopteran pheromones are classified into four types based on their chemical structure [3]. The SPCs of *E. pseudoconspersa* have been identified to be 10Me14Me-15:iBu and 14Me-15:iBu, which belong to the Type III pheromone group. Our previous study demonstrated that 10Me14Me-15:iBu has a stereogenic center and that the (*R*)-enantiomer has a significantly stronger biological activity in male moths than the (*S*)-enantiomer [19]. The proteins involved in Type III pheromone detection remain unknown. To obtain sequence data on *E. pseudoconspersa*’s olfactory perception, especially for pheromone detection, we sequenced and analyzed tissue transcriptome profiles. In total, 183.38 Gb of transcriptome data and 295,509 unigenes were obtained. After annotation, 161 candidate olfactory genes were identified, including 27 EpseCSPs, 39 EpseOBPs, 28 EpseIRs, and 67 EpseORs. The number of candidate olfactory genes was higher than reported in previous studies on the transcriptomes of other lepidopteran insects, such as *H. armigera* [20], *Spodoptera litura* [21], *Spodoptera exigua* [22], *Chilo suppressalis* [23], *Scopula subpunctaria* [24,25], and *Ectropis grisescens* [26]. This might have been because large amounts of high-quality transcriptome data were obtained in the current study.

OBPs are liaisons between the external environment and ORs [27,28]. Olfactory detection begins when odor molecules enter the olfactory sensilla through cuticular pores. To reach the ORs, the most strongly hydrophobic odor molecules are bound and solubilized by OBP and transported through the sensillum lymph surrounding the dendrites [29]. According to the number and pattern of cysteines, insect OBPs can be categorized into three classes (“classic”, “minus-C”, and “plus-C”) [30]. The lepidopteran PBP/GOBP genes, which belong to classic OBPs, are lepidopteran-specific monophyletic subgroups of insect OBPs [31]. The 39 EpseOBPs were divided into three classes: classic, minus-C, and plus-C. Of them, four EpsePBPs and two EpseGOBPs were identified. These six genes were scattered in different clades of the PBP/GOBP subgroup with genes of Type I and II SPC species. Gene expression studies showed that the *PBPs* of Type I and II pheromone moth species were primarily expressed in the antennae of male adults. *EpsePBP1*, *EpsePBP2*, and *EpsePBP3* were more abundant in male antennae than in female antennae, with higher FPKM values than other *EpseOBPs*. These results suggest that moth PBPs are conserved among moth species with different pheromones.

Other OBPs and CSPs have been detected in trichoid sensilla, which are sensitive to sex pheromones. For example, GOBPs showed a high binding affinity for Type I and II SPCs [32], while HarmCSP6 and SinfCSP19 showed high binding affinities for the SPCs of *H. armigera* and *Sesamia inferens*, respectively [33]. *EpseGOBP1* and *EpseGOBP2* were mainly expressed in the antennae, with higher FPKM values than most *EpseOBPs*. In addition to *EpsePBPs*, *EpseOBP13* was the most common male antenna-biased *EpseOBP*. Its FPKM value was similar to *EpsePBP1* and *EpsePBP2*. Therefore, EpseOBP13 might play important roles in the detection of chemical signals from the external environment, particularly in the binding of SPCs in *E. pseudoconspersa*. A few antenna-biased EpseOBPs and EpseCSPs, particularly those highly expressed in female antennae, may be involved in host plant volatile perception [34,35].

The discovery of ORs tuned to odors in insects is a major breakthrough in our understanding of the olfactory molecular mechanisms of insects. Insect ORs function as complexes composed of a ligand-specific OR and co-receptors (Orcos) [36,37]. Type I pheromones account for 75% of all known moth SPCs [9,11]. Consequently, the vast majority of lepidopteran PRs that have been identified and characterized to date are specifically tuned to Type I SPCs [38]. These PRs mostly fall into the monophyletic clade of ORs, named the “PR clade”. ObruOR1, a member of the “PR clade”, was a PR for Type II pheromones, which was tuned to the Type II pheromones [39]. A “Type II PR clade” was observed between *E. grisescens*, *E. obliqua*, and *S. subpunctaria* [24,40]. One PR of the clade, EgriOR31, was tuned to the Type II SPCs (Z,Z,Z)-3,6,9-octadecatriene and (Z,Z)-3,9-6,7-epoxyoctadecadiene [26]. To date, no PRs have been reported for Type III SPCs. *Euproctis pseudoconspersa* is a typical Type III SPC moth. In the current study, no EpseORs fell within the “PR clade”, whereas five EpseORs were distributed in the “Type II PR clade”. In support of their role as receptors for female-released SPCs, PRs were mainly expressed in the trichoid sensilla of male antennae. All five *EpseORs* were significantly more abundant in male than female antennae. Consequently, these five EpseORs may function as PRs. *EpseOR13* was the most abundant *EpseOR* in male antennae, with significantly higher FPKM values.

In recent years, many studies have revealed that moth PRs for different types of SPCs (Types 0, I, and II) and those for the same type do not fall into a common clade within the moth OR family. Different PR lineages of lepidopterans appear to have evolved independently. For example, SlitOR5 from *Spodoptera littoralis* is tuned to the Type I pheromone (Z,E)-9,11-tetradecadienyl acetate; however, it does not belong to the “PR clade” [41]. EsemOR3 and EsemOR4 from *Eriocrania semipurpurella* are PRs for Type 0 pheromones that do not distribute in the previously defined lepidopteran “PR clade” [42]. In the current study, we identified an independent group formed by five EpseORs without orthologs from other lepidopteran insects in the phylogenetic analysis. Three EpseORs of the group, *EpseOR14*, *EpseOR32*, and *EpseOR40*, showed significantly higher FPKM values in male antennae than in female antennae. *EpseOR32* and *EpseOR14* were the second and third most abundant *EpseORs* in the male antennae of *E. pseudoconspersa*, respectively. Therefore, EpseOR32 and EpseOR14 might be potential PRs in *E. pseudoconspersa* SPC detection.

Insects rely on plant volatiles to locate appropriate hosts for feeding and oviposition. ORs play an important role in the detection of plant volatile compounds. For example, *Lema daturaphila*-induced plant volatile α-copaene can elicit *Manduca sexta* oviposition, and OR35 is involved in detecting α-copaene [43]. OR47 is primarily tuned to nonanal pheromones, which are attractive to virgin *Spodoptera frugiperda* females [44]. Seven EpseORs were significantly more highly expressed in female antennae than in male antennae. Of them, EpseOR25, EpseOR59, and EpseOR64 had values higher than 10. Therefore, these genes may be involved in the detection of plant volatiles. In addition to ORs, antennal IRs mediate the detection of certain odorants, including organic acids, amines, and aldehydes. IR8a and IR25a are IR co-receptors that form ligand-gated ion channels with other odorant-tuned IRs. Genome editing and behavioral analyses have demonstrated that IR8a is essential for acid-mediated fecal avoidance in ovipositing *M. sexta* [8]. In the current study, EpseIR7 and EpseIR20 were distributed within the IR8a/IR25a clades. *EpseIR7* was the most abundant *EpseIR* in *E. pseudoconspersa*, supporting its role as a co-receptor for other IRs.

## 4. Materials and Methods

### 4.1. Insect Rearing and Tissue Collection

Individual *E. pseudoconspersa* larvae were first collected from a tea plantation of Fujian Tianhu Tea Industry Co., Ltd. (Fuding, Ningde, China). Larvae were reared on fresh tea shoots in enclosed nylon mesh cages (50 × 50 × 50 cm) at a temperature of 25 ± 1 °C and a humidity of above 65% under a 14:10 h (L:D) photoperiod. After pupation, male and female pupae were separated and maintained in separate cages. After emergence, the antennae, pheromone glands, heads, and bodies of 20 mature virgin female adults (2 d old) and the antennae, heads, and bodies of 20 mature virgin male adults (2 d old) were collected for transcriptome sequencing, performed in three replicates. The same tissues were collected from 2 d old virgin moths for quantitative real-time PCR (qPCR), performed in three replicates. All excised tissues were immediately frozen and stored at −80 °C until RNA isolation.

### 4.2. cDNA Library Construction, Illumina Sequencing, Assembly, and Annotation

Total RNA was extracted using the TRIzol reagent (Invitrogen, Carlsbad, CA, USA). RNA quality was assessed using gel electrophoresis and a NanoDrop One spectrophotometer (NanoDrop, Wilmington, DE, USA). cDNA library construction and Illumina sequencing of the qualified RNA samples were performed at Novogene Co., Ltd. (Beijing, China). The Illumina HiSeq platform (Illumina, San Diego, CA, USA) was used for sequencing and library preparation, and paired-end reads were generated. After removing reads containing adapters, poly-N, and low-quality reads, transcriptome assembly was performed based on paired-end reads using the short-read assembly program Trinity. The assembled transcripts were annotated using the Nr, Nt, Pfam, KOG/COG, Swiss-Prot, KEGG Ontology, and GO databases.

### 4.3. Identification and Phylogenetic Analysis of Olfactory Gene Families

Putative olfactory genes were annotated by searching against the Nr database using BLASTX with a cut-off *e*-value of 10^−5^. All putative olfactory genes were confirmed using the NCBI blastx. The ORFs of *E. pseudoconspersa* olfactory genes were predicted using ORF Finder (https://www.ncbi.nlm.nih.gov/orffinder/, accessed on 12 August 2024). The signal peptides of EpseOBPs were predicted using SignalP 6.0 (https://services.healthtech.dtu.dk/services/SignalP-6.0/; accessed on 15 May 2024). For the phylogenetic analysis, OBPs, IRs, and ORs were aligned with those of other lepidopteran species using MAFFT (E-INS-I parameter). Maximum-likelihood phylogenetic trees were constructed using PhyML 3.1 with the LG substitution model [45]. Finally, the trees were viewed and group-edited using FigTree v1.4.2.

### 4.4. Abundance Analysis

Transcript abundance was calculated using the FPKM method using the following equation:

$$FPKM\left(A\right)=\frac{{C\times 10}^{6}}{\frac{N\times L}{10^{3}}}\;,$$

where *FPKM*(*A*) represents the abundance of transcript *A*, *C* is the number of fragments uniquely aligned to transcript *A*, *N* is the total number of fragments uniquely aligned to all transcripts, and *L* is the number of bases in transcript *A*. The differential expression analysis of the two groups was performed using the DESeq R package (1.10.1) [46]. The *p*-values were adjusted using Benjamini and Hochberg’s approach to control the false discovery rate. Genes with an adjusted *p*-value < 0.05, observed using DESeq, were considered differentially expressed.

### 4.5. qPCR Validation

The tissue expression patterns of 18 olfactory genes, including 4 EpesOBPs and 14 EpseORs, were determined using qPCR to validate the transcript abundance analysis. qPCR was conducted on a Roche LightCycler 480 detector (Stratagene, La Jolla, CA, USA) using ChamQ SYBR qPCR Master Mix (Vazyme, Nanjing, China). Primers were designed using Beacon Designer 7.7 based on the nucleotide sequences obtained from the transcriptome data (Table S5). The reaction was performed as follows: 30 s at 95 °C, followed by 40 cycles of 95 °C for 10 s, and 60 °C for 30 s. Each reaction had three independent biological replicates and was repeated three times (technical replicates). The geometric mean of multiple reference genes was validated as an accurate normalization factor by analyzing publicly available microarray data [47]. Therefore, two genes—*guanine glyceraldehyde-3-phosphate dehydrogenase* (GAPDH) and *ribosomal protein S3* (rpS3)—were selected as reference genes for the qPCR analysis. The geometric mean of GAPDH and rpS3 was calculated using Excel and selected as an internal control for the qPCR analysis. The relative gene expression levels were calculated using the comparative 2^−∆∆CT^ method [48].

## 5. Conclusions

We sequenced the tissue transcriptome profile of *E. pseudoconspersa*, a typical Type III SPC moth species. We obtained 1.22 billion clean reads with 183.38 Gb of data. After assembly and annotation, 295,509 unigenes were identified, and 169,459 were annotated. Among these unigenes, we identified 161 olfactory genes (27 EpseCSPs, 39 EpseOBPs, 28 EpseIRs, and 67 EpseORs) and conducted phylogenetic and tissue abundance analyses. Four EpsePBPs and eight EpseORs were identified as candidates for Type III sex pheromone detection. We observed that the PRs of *E. pseudoconspersa* did not fall within the PR clade, indicating that the PRs for different SPC types of lepidopteran insects could have independently evolved. Additional functional and evaluation analyses are needed to verify this conjecture.

## Data Availability

Data is contained within the article or Supplementary Material.

## Supplementary Materials

The following supporting information can be downloaded at https://www.mdpi.com/article/10.3390/ijms26041405/s1.
