# Supplementary material for: Transcriptome Profiling of Euproctis pseudoconspersa Reveals Candidate Olfactory Genes for Type III Sex Pheromone Detection
Source: Int J Mol Sci. 2025 Feb 7;26(4):1405. doi: 10.3390/ijms26041405 (PMC11855508; doi:10.3390/ijms26041405)
Supplement: Supplementary file 1 [file ijms-26-01405-s001.zip › Table S1 and Table S2.pdf]

Table S1. ***Euproctis pseudoconspersa*** **transcriptome assembly.**

	Transcripts	unigenes
Min length	201	201
Mean length	754	937
Median length	318	419
Max length	88,395	88,395
N_{50}	1,667	2,002
N_{90}	259	319
Total nucleotides	306,177,433	276,804,352

Table S2. ***Euproctis pseudoconspersa*** **transcriptome annotation.**

Database	Number of Unigenes	Percentage
Annotated in Nr	106,311	35.97
Annotated in Nt	119,339	40.38
Annotated in KO	3,658	1.23
Annotated in SwissProt	92,048	31.14
Annotated in Pfam	101,108	34.21
Annotated in GO	101,108	34.21
Annotated in KOG	33,031	11.17
Annotated in all Databases	1,849	0.62
Annotated in at least one Database	169,459	57.34
Total Unigenes	295,509	100
